# Differential transcription of virulence genes in *Aggregatibacter actinomycetemcomitans* serotypes

**DOI:** 10.3402/jom.v5i0.21473

**Published:** 2013-10-22

**Authors:** Josely Emiko Umeda, Priscila Larcher Longo, Maria Regina Lorenzetti Simionato, Marcia Pinto Alves Mayer

**Affiliations:** 1 Department of Microbiology, Institute of Biomedical Sciences, University of São Paulo, São Paulo, Brazil

**Keywords:** *Aggregatibacter actinomycetemcomitans*, gene expression, epithelial cell, infection

## Abstract

**Background:**

*Aggregatibacter actinomycetemcomitans* serotypes are clearly associated with periodontitis or health, which suggests distinct strategies for survival within the host.

**Objective:**

We investigated the transcription profile of virulence-associated genes in *A. actinomycetemcomitans* serotype b (JP2 and SUNY 465) strains associated with disease and serotype a (ATCC 29523) strain associated with health.

**Design:**

Bacteria were co-cultured with immortalized gingival epithelial cells (OBA-9). The adhesion efficiency after 2 hours and the relative transcription of 13 genes were evaluated after 2 and 24 hours of interaction.

**Results:**

All strains were able to adhere to OBA-9, and this contact induced transcription of *pgA* for polysaccharide biosynthesis in all tested strains. Genes encoding virulence factors as Omp29, Omp100, leukotoxin, and CagE (apoptotic protein) were more transcribed by serotype b strains than by serotype a. *ltxA* and *omp29*, encoding the leukotoxin and the highly antigenic Omp29, were induced in serotype b by interaction with epithelial cells. Factors related to colonization (*aae*, *flp*, *apaH*, and *pgA*) and *cdtB* were upregulated in serotype a strain after prolonged interaction with OBA-9.

**Conclusion:**

Genes relevant for surface colonization and interaction with the immune system are regulated differently among the strains, which may help explaining their differences in association with disease.

The oral species *Aggregatibacter actinomycetemcomitans* is a recognized periodontopathogen. Genome analysis revealed that this species is distributed in two major groups correlated with serotype-specific polysaccharide antigens: serotypes: a, d, e, and f, and serotypes b and c, which may help to explain their differences in virulence. About 2,000 genes were included as the core genes in this bacterium, whereas 16.7–29.4% of the genome belongs to the flexible gene pool (1).

Serotype b is frequently associated with aggressive periodontitis (2), and subjects harboring the serotype b JP2 clone are at a higher risk of developing aggressive periodontitis than those colonized by ‘non-JP2 clones’ strains (3). The main characteristic of the virulent clone is the expression of high levels of leukotoxin (4), but other genotypes within the serotype b have also been associated with aggressive periodontitis (5). Serotypes a and c are associated with health (2), although the latter may also be found in periodontitis-affected patients (6). Certain phenotypic and genotypic features have been associated with *A. actinomycetemcomitans* serotypes (7), but little is known on the expression of core genes and its effects on the virulence potential of different lineages.

*A. actinomycetemcomitans* expresses virulence factors, which promote adhesion to oral surfaces, suppress or inactivate the host immune response and induce inflammation and tissue destruction. The expression of fimbriae and polysaccharide mediates formation of dense biofilms (8, 9). Adhesins, such as Aae and Omp100, play a significant role in the interaction with gingival epithelial cells (10, 11), whereas the highly immunogenic outer membrane protein Omp29 (12) and the tetraphosphatase ApaH (13) are associated with invasion of non-phagocytic cells. Leukotoxin induces apoptosis not only in neutrophils and macrophages of humans and Old World primates (5) but also in endothelial cells (14). This organism's cytotoxic effects are also mediated by the cytolethal-distending toxin, which is involved in damage of the epithelial cells barrier (15) and in the interaction with the immune system (16, 17).

Since most virulence-associated genes are part of the core genome of *A. actinomycetemcomitans*, this study tested the hypothesis that differences in the virulence potential of *A. actinomycetemcomitans* strains may be partly due to differences in their transcription profile.

## Material and methods

### Bacterial strains and culture conditions

Reference strains ATCC 29523 (serotype a) and JP2 and SUNY 465 (serotypes b) were used. Strains were subcultured in Tryptic soy agar or broth supplemented with 0.6% of yeast extract (TSYE), at 37°C in 10% CO_2_. All strains exhibited the smooth phenotype when grown in agar plates, and were non-aggregative when grown in broth. The colonies of *A. actinomycetemcomitans* grown in TSYE agar were inoculated in TSBYE and incubated for 8 hours. The suspension was adjusted to an OD_495 nm_∼1, diluted 1:40 in the same broth, and incubated for 7 hours, corresponding to exponential growth phase/mid log. The cell density was adjusted to an OD_495 nm_∼0.2 corresponding to 3×10^8^ CFU/ml.

### Epithelial cell culture

Immortalized gingival epithelial cells (OBA-9) (12), gently given by Dr. Shinya Murakami (University of Osaka, Japan), were cultured in serum-free keratinocyte medium containing insulin, epidermal growth factor, and fibroblast growth factor (KSFM-Invitrogen, Carlsbad, CA), supplemented with 100 µg/ml streptomycin and 100 U/ml penicillin (Sigma, St. Louis, MO) at 37°C in 5% CO_2_.

### Adhesion assay

OBA-9 cells were inoculated in 24-well tissue culture plates (Corning Inc., Corning, NY) and incubated to reach a semiconfluent monolayer (∼3 x 10^5^ cells/well) in KSFM. Prior to infection, the wells were washed with phosphate-buffered saline (PBS) (pH 7.5, 0.8% NaCl). Overnight bacterial cultures were inoculated in TSYE broth to reach exponential growth phase/mid log, harvested by centrifugation, resuspended in antibiotic-free KSFM and inoculated in OBA-9 cells monolayers at a multiplicity of infection (MOI) of 3,000:1 (bacteria: eukaryotic cell) (18). Plates were centrifuged at 593×g/10 min and incubated for 2 and 24 hours. Non-adherent bacterial cells were removed by washing with PBS. The remaining bacterial cells firmly attached to and/or invading the epithelial cells were evaluated for gene expression after interaction with epithelial cells. Controls consisted of bacterial suspensions in KSFM without the addition of OBA-9. The percentage of adherent bacterial cells after 2 hours incubation was determined after trypsin treatment of the co-cultures by CFU evaluation.

### Gene transcription analysis

The co-cultures, after 2 hours (B) and 24 hours (D) of interaction, were washed with PBS for removal of non-adherent bacteria, and submitted to RNA extraction using Trizol (Invitrogen). Bacterial suspensions in KSFM incubated for 2 and 24 hours (A and C) were used as controls. After chloroform extraction, RNA was precipitated with isopropanol and washed with 70% ethanol. Genomic DNA was removed by DNase digestion. First-strand synthesis was performed with 1 µg of RNA, using the First-Strand Synthesis System for RT-PCR with random hexamer primers (Super Script III, Gibco, Grand Island, NY). Transcription of 13 genes was evaluated by real-time polymerase chain reaction (qPCR), using the primers described in Table 1. Controls with no addition of RNA, addition of RNA and no addition of reverse transcriptase (used as control for the DNase treatment) were included, and their products were used as parallel control samples in qPCR.

**Table 1:** Oligonucleotide primers used for RT-qPCR

Gene	Nucleotide sequence (5’–3’)	Product (pb)	Reference
*gapdh*	CCCAAAACATCATCCCATCTTCGGAACACGGAACGCCATAC	60	18
*ltxA*	TTGTCGCAAGTGCCATAGTTATCCACTAGCCCCATGGCAACGGTAGAA	193	This study, 22
*flp*	TCAAAGCAATCGAAGCAATCGCAATAGCGATCAAACCGTA	82	This study, 22
*aae*	GGTTTTAGGCGGCACATTTATGCTTGACCAACCATAACCA	152	This study, 22
*apaH*	CACCTTGGTTTGCCTTGGATATGTCTTCCCAACGTAGCATG	159	This study, 22
*omp29*	TCTCAACAAGCCATCTCTGCCGACCTTTAACTACGTCGCA	80	This study, 22
*omp100*	ATCTTCAAGCCAAAACATCAAGGCTGCCGACATTAT	169	11
*cdtB*	CAACAACACAATTCCAACCCGGCGATACCTGTCCATTCTT	94	This study, 22
*vapA*	CGAAATTATGGCTGGGTATGCAATGGTTGGATGTTGAATACGG	60	31, 22
*orf* 859	CAATCTCACCCAAGCCCTACGCGGCGGAAATATAGAAACTG	83	31
*vppA*	GGTTACCGGTGGAGTTCGCGGGTCGTAATCGTTTGA	190	This study, 22
*cagE*	TGGATTGGGACAAGTGAACATACAAAGCCATAAGAGAAAT	190	This study, 22
*emaA*	CTGCAGCAACCGGGGATTATAATGGATTGGTTGCCTTTAG	110	This study, 22
*pgA*	GACGGTGATGCGGTATTGGGACCGATGATGGAGCTGAA	160	9

The reaction consisted of 10 µl SYBR Green PCR Master Mix (Biotools Inc., QuantiMix EASY SYG Kit, Madrid, Spain), 200 nM of each primer, 100 ng of cDNA, 3–4.5 mM MgCl_2_ in 20 µl of final volume. The housekeeping gene *gapdH* (*orf1383*) was used to normalize mRNA levels (18). Reactions were performed in iQ 5 Bio Rad thermocycler (Bio Rad, Hercules, CA) linked to software (IQ-5 Real Time PCR Detection System), in 48 cycles with 95°C/10 sec, 50°C/1 min (18) and 80–83°C for 6 sec.

Melting curve analysis was carried out with 10 sec of 100 repetitions by increasing the annealing temperature by 0.4°C per step from 55 to 95°C (18). All samples were examined in triplicate, with parallel control samples without cDNA, and internal controls of *gapdH* amplification in each experiment. Three independent RNA samples were analyzed. The relative expression of the studied genes was analyzed with REST2005 Beta V1.9.10 (19) (Corbett Life Science, Sydney, Australia), by using the following formula:

$$\text{Mean ratio of gene expression}={\left(E_{\text{target}}\right)}^{\Delta \text{C}{\text{P}}_{\text{target}\left(\text{mean control}-\text{mean sample}\right)}}/{\left(E_{ref}\right)}^{\Delta \text{C}{\text{P}}_{\text{ref}\left(\text{mean control}-\text{mean sample}\right)}}$$

The relative expression ratio of target gene was computed based on its real-time PCR efficiencies (*E*) and the crossing point (CP) difference (▵) of an unknown sample versus a control (▵cp mean control − mean sample). The target gene expression was normalized by a non-regulated reference gene expression, *gapdH*.

The choice of *gapdH* as control gene was based on our previous observation, by multiple RT-PCR assays, that this gene is expressed at the same level in *A. actinomycetemcomitans* cells grown in culture medium at different conditions (22). Furthermore, quantitative RT-PCR has also been done with some of these samples using 16S rRNA primers as internal control (data not shown), and the results did not differ significantly from those with *gapdH* as the control.

The effect of interaction of bacteria with epithelial cells (B or C) was evaluated for each strain by comparison with data obtained at control condition without cell interaction (A or C). In addition, the relative gene expression of strains ATCC 29523 and SUNY 465 was compared to the data obtained for strain JP2 at each studied condition (A, B, C and D). Differences were determined by ANOVA-TUKEY. Statistically significant differences were considered when *p* was <0.05. When significant differences were determined, fold changes in relation to control condition were represented as follows:

Fold changes=Mean ratio of gene expression at the tested condition/Mean ratio of gene expression at control.

When significant differences were determined, fold changes of strains SUNY 465 and ATCC 29523 in relation to strain JP2 at the same condition were determined as follows:

Fold changes=Mean ratio of gene expression for the tested strain/Mean ratio of gene expression for JP2.

## Results

All tested strains were able to adhere to OBA-9 cells after 2 hours of interaction. The percentages of adherent cells in relation to the initial inocula were (average±SD): 0.31%±0.14 for ATCC 29523%3B 0.41%±0.014 for JP2; and 1.2%±0.62 for SUNY465.

The 13 tested genes were detected in genomes of the three strains (data not shown) and were transcribed by strain JP2; thus, the relative transcription of each gene in JP2 was used for comparison. Mean relative expression ratios after interaction with epithelial cells and the *in vitro* control condition are shown in Table 2. Interaction with epithelial cells led to differences in gene expression in all studied genes, except for *cagE*. In fact, *cagE* was only transcribed by strain JP2 in control and by SUNY 465 after interaction with epithelial cell. *pga* was upregulated after interaction with epithelial cells in all strains (Table 2). *orf 859* was shown to be upregulated only in ATCC 29523 after 24 hours interaction with epithelial cells, but exhibited no detectable transcripts in the control condition.

**Table 2:** Effect of interaction with epithelial cells on the relative gene expression of different *A. actinomycetemcomitans* isolates after 2 and 24 h of incubation

Relative gene expression after interaction of bacteria with epithelial cells (B or D) in relation to control at 2 and 24 h incubation (A or C)												

Strain	JP2				SUNY 465				ATCC 29523			
			
Condition	2h [B/A]		24h[D/C]		2h [B/A]		24h[D/C]		2h [B/A]		24h[D/C]	
						
Gene	Relative gene expression mean±sd	Fold changes	Relative gene expression mean±sd	Fold changes	Relative gene expression mean±sd	Fold changes	Relative gene expression mean±sd	Fold changes	Relative gene expression mean±sd	Fold changes	Relative gene expression mean±sd	Fold changes
*flp*	0.36±0.25/0.25±0.05		**1.95±0.57/0.53±0.25**	3.68	0.11±0.03/0.16±0.01		0.068±0.02/0.10±0.00		0.30±0.07/0.27±0.02		**50.41±18.66/0.002±0.00**	25,205
*aae*	0.54±0.28/1.048±0.52		0.65±0.27/0.42±0.09		**1.01±0.08/0.39±0.03**	2.6	0.96±0.31/2.07±0.19		**0.43±0.03/0.001±0.00**	430	**19.23±4.58/0,004±0.00**	4,807.5
*apaH*	**1.06±0.015/2.22±0.67**	0.48	0.47±0.24/0.62±0.19		0.00 ±0.00/0.42±0.09		**0.00±0.00/0.53±0.09**	#	0.18 ±0.09/0.16±0.03		**8.74±1.43/0.003±0.00**	2,913.33
*pgA*	**2.09±0.65/0.23±0.04**	9.09	**2.43±0.26/1.03±0.12**	2.36	**23.94±5.56/0.30±0.10**	79.8	**24.19±2.21/0.66±0.14**	36.7	**3.81±2.12/0.02±0.00**	190.5	**43.39±3.98/0.15±0.13**	289.27
*emaA*	0.204±0.04/0.27±0.00		**0.23±0.01/0.09±0.01**	2.56	**0.019±0.00/0.052±0.01**	0.37	**0.06±0.02/0.11±0.03**	0.55	**0.01±0.00/0.0±0.00**	#	0.00±0.00/0.00±0.00	
*vppA*	5.64±0.65/9.41±1.68		3.94±1.11/7.08±0.71		**0.88±0.18/2.96±0.51**	0.30	**2.04±1.15/10.75±1.87**	0.19	**0.54±0.21/0.005±0.00**	108	**71.49±10.66/0.008±0.01**	8,936.25
*cdtB*	6.54±1.23/9.95±0.62		4.21±1.37/4.75±0.69		**0.89±0.69/2.81±0.83**	0.32	**1.56±0.75/4.98±0.90**	0.31	0.07±0.01/0.34±0.04		**68.09±21.18/0.004±0.00**	17,022.5
*vapA*	3.57±0.74/4.54±0.19		**3.66±0.58/8.07±1.85**	0.45	**6.16±1.58/1.22±0.04**	5.05	8.46±1.51/5.80±0.88		**2.76±0.27/0.01±0.00**	276	**29.73±3.44/0.04±0.00**	743.25
*omp29*	23.54±1.12/20.79±2.84		**46.40±16.10/171.14±23.16**	0.27	**1.25±0.49/0.20±0.01**	6.25	**0.33±0.28/5.54±1.77**	0.06	0.00±0.00/0.00±0.00		0.00±0.00/0.00±0.00	
*omp100*	0.16±0.04/0.07±0.04		0.25±0.01/0.37±0.05		**0.50±0.29/0.005±0.00**	100	**1.99±0.53/0.16±0.02**	12.45	**0.42±0.16/0.001±0.0**	420	**0.08±0.01/0.017±0.01**	4.71
*ltx*	10.76±4.72/9.43±2.39		2.06±0.53/3.88±1.92		2.36±0.27/2.80±0.72		**2.54±0.41/0.56±0.15**	4.54	**0.36±0.08/0±0.00**	#	**0.125±0.06/0.004±0.00**	31.25
*orf59*	0.04±0.01/0.11±0.00		0.03±0.02/0.07±0.00		0.15±0.08/0.18±0.03		0.06±0.00/0.10±0.02		0.00±0.00/0±0.00		**0.39±0.10/0.00±0.00**	#
*cagE*	1.18±0.08/0.223±0.08		0.07±0.02/0.15±0.04		0.00±0.00/0.00±0.00		0.12±0.07/0.07±0.03		0.00±0.00/0±0.00		0.00±0.00/0.00±0.00	

The data are shown as mean relative gene expression in strains JP2, SUNY 465 and ATCC 29523 after interaction with epithelial cells in relation to control condition, for 2 h [B/A] and 24 h [D/C]. Fold changes are only shown when differences in relative gene expression between control and test conditions were statistically significant.

Bold – significant difference between control (A or C) and test (interaction with epithelial cell – B or D) (Anova-Tukey p <0.05).

# no detectable transcripts at control condition or test condition, significant difference between control and test.

Fold changes=relative gene expression tested condition (with epithelial cell interaction)/relative gene expression control.

The mean relative expression ratios for each gene at each studied condition were also compared among the three strains. The levels observed for strain JP2 were considered as controls, and data provided as fold changes in relation to JP2 at each condition (Table 3). At the control, without interaction with epithelial cells, genes such as *aae*, *omp100*, *emaA*, and *pgA* were not transcribed or transcribed in very low levels by ATCC 29523 (serotype a), when compared to JP2. In addition, genes associated with host evasion such as *ltxA*, *omp29*, *cdtB*, and *cagE* were less transcribed by this strain when compared to JP2, and most of them kept being transcribed in very low levels after 2 hours interaction with epithelial cells. However, after 24 hours of interaction, several genes such as *flp*, *aae*, *apaH*, *pgA*, and *cdtB* were upregulated in the serotype a strain (Table 2), surpassing the mRNA relative levels encountered for JP2 (Table 3).

**Table 3:** Fold changes of relative gene expression of strains ATCC 29523 and SUNY 465 in relation to strain JP2 at the same condition

	Fold changes of relative gene expression in relation to JP2							
	
	2 h in culture medium [A]		2 h interaction epithelial cells [B]		24 h in culture medium [C]		24 h interaction epithelial cells [D]	
				
Gene	ATCC 29523	SUNY 465	ATCC 29523	SUNY 465	ATCC29523	SUNY 465	ATCC 29523	SUNY 465
*flp*		0.64		0.31	0.004	0.19	25.85	0.035
*aae*	0.001	0.37			0.01	4.93	29.58	
*apaH*	0.07	0.19	0.17	0	0.01		18.60	0
*pgA*	0.09	1.30		11.45	0.15		17.86	9.95
*emaA*	0	0.19	0.05	0.09	0		0.00	
*vppA*	0.01	0.31	0.10	0.16	0.01		18.14	
*cdtB*	0.03	0.28	0.01	0.14	0.01		16.17	0.37
*vapA*	0.00	0.27		1.73	0.00		8.12	2.31
*omp29*	0	0.01	0.01	0.053	0	0.03	0	0.01
*omp100*	0.01	0.07		3.13	0.05	0.43	0.32	7.96
*ltxA*	0	0.30	0.03	0.22	0.00	0.14	0.061	
*orf859*	0	1.64	0	3.75	0		13	
*cagE*	0	0	0	0	0		0	

Fold changes are only shown when differences in relative gene expression between tested strain and control strain (JP2) were statistically significant (Anova-Tukey *p*<0.05).

Fold changes= mean ratio of relative gene expression of tested strain/mean ratio of relative gene expression of strain JP2 at each studied condition (A, B, C, or D).

Most studied genes, except for *pgA* and *orf859*, were transcribed in lower levels by the serotype b strain SUNY 465 when compared to JP2 in the control for 2 hours (Table 3). Interaction of this strain with epithelial cells resulted in increased mRNA levels of *aae*, *omp100*, *ltxA*, *vapA*, and *omp29* (Table 2). Indeed, long-term interaction (24 hours) with epithelial cells resulted in similar or higher levels of transcription of most virulence-associated genes in SUNY 465 when compared to JP2, except for *omp29* (Table 3). On the other hand, the genes encoding colonization factors were several folds less transcribed in the two serotype b strains than in the serotype a after prolonged exposure to epithelial cells (Table 3).

*ltxA*, encoding the leukotoxin, was transcribed in lower levels by ATCC 29523 and SUNY 465 when compared with JP2 in the controls (A and C). However, interaction with epithelial cells upregulated its transcription in both strains (Table 2), and *ltxA* mRNA levels were similar in SUNY 465 and JP2 after long-term interaction with epithelial cells (Table 3).

The fimbriae subunit encoding gene, *flp*, was transcribed in low levels in the strains, as expected for smooth isolates. However, prolonged contact with epithelial cells resulted in upregulation of *flp* in ATCC 29523 (serotype a).

Genes within the *cdt* operon, *cdtB* and *vppA* (20), were transcribed in low levels after *in vitro* growth by ATCC 29523 (Table 3) and were upregulated after prolonged incubation with eukaryotic cells.

## Discussion

Epithelium is the first physical barrier limiting bacterial penetration into tissues in the oral cavity. Using a co-culture model, we had shown that epithelial cells respond to *A. actinomycetemcomitans* insult by altering the transcription of selected genes, especially those involved in tissue remodeling and bone resorption (21). Bacteria may also sense the environment and change their gene expression profiles, due to signal transduction systems, increasing their fitness to the challenges promoted by the immune response. In a prior study, we have shown that *A. actinomycetemcomitans* senses the environment and changes gene transcription according to the phase of the *in vitro* growth (22).

In this study, all strains were able to adhere to gingival epithelial cells although their adhesion efficiency varied as shown previously (10). So, it was possible to evaluate the transcription profiles of the three isolates when they were firmly attached to, and/or invading the epithelial cells, condition that we have denominated as bacterial–epithelial cells interaction.

As expected, the leukotoxin encoding gene, *ltxA*, was several folds more expressed in JP2 than in the other strains (4). However, prolonged interaction with epithelial cells upregulated *ltxA* transcription in SUNY 465, but not in JP2 (Tables 2 and 3). Other studies suggested that serotype b isolates not belonging to the JP2 clone may express *in vivo* levels of leukotoxin equivalent to the JP2 clone (23), and our data indicate the need of further investigation on the leukotoxin levels *in vivo*.

*flp*, encoding the fimbrial subunit, was upregulated in ATCC 29523 after prolonged interaction with epithelial cells, suggesting that the fimbriated phenotype may be restored under certain circumstances. The conversion to the smooth phenotype was associated with mutations in the *tad* locus (24), but environmental factors such as pH, temperature, oxygen, and iron concentrations have also been shown to influence fimbriae expression (25). In addition, an early study indicated that adherence accompanied a smooth to rough phenotype shift (26). Thus, the increase in *flp* relative expression after cell interaction may indicate that contact with epithelial cells may positively influence fimbriae expression.

Cytolethal-distending toxin (CDT) targets a variety of cells including immune system cells and modulates the immune response by inducing apoptosis of non-proliferative monocytic cells and T lymphocytes (16, 17). mRNA levels of *cdt* were low in serotype a reference strain at the control condition (Table 3), supporting our previous finding that this strain is not as cytotoxic as JP2 (6, 27). However, *cdtB* was upregulated after prolonged cell exposure in the serotype a strain, suggesting that it may be able to produce high levels of CDT under certain conditions.

Transcriptions of *aae* and *apaH*, involved in adherence (10) and invasion to epithelial cells (13), respectively, were upregulated in ATCC 29523 after prolonged interaction with eukaryotic cells. Upregulation of *apaH* was also observed for strain SUNY 465 after prolonged interaction with epithelial cells (24 hours), whereas it was downregulated in JP2 after 2 hours of interaction (Table 2). ApaH is a diadenosine tetraphosphatase, member of the nudix hydrolase family. Deletion of the gene encoding Ap_4_A hydrolases from pathogenic bacteria reduces their ability to invade mammalian cells, which is possibly associated to a role of Ap_4_A as a regulator of mRNA degradation (28). Thus, the intense upregulation of *apaH* observed in strain ATCC 29523 after 24 hours of interaction with epithelial cells may have resulted in other changes in the transcription profile of the bacterial cell.

Furthermore, *omp100*, encoding the highly immunogenic autotransported protein involved in adherence to epithelial cells (11), was transcribed at low levels by strain ATCC 29523 and SUNY 465 after *in vitro* incubation (Table 3) when compared with JP2, and it was upregulated after interaction with epithelial cells. Indeed, *omp100* mRNA levels in SUNY 465 surpassed the levels observed for JP2 (Table 3).

On the other hand, *omp29* was transcribed at very low levels by ATCC 29523, and SUNY 465, when compared to JP2 in all studied conditions (Table 3). *A. actinomycetemcomitans* Omp29 belongs to the OmpA family, abundant outer membrane proteins conserved in gram-negative pathogens (29). This protein is associated with virulence in experimental animal models and with invasion of non-phagocytic cells (12, 29) and the higher *omp29* transcript levels of JP2 may represent an additional characteristic in its pathogenic potential when compared to the others.

Thus, transcription analysis of *omp100*, *aae*, and *omp29* suggests that the strategies promoted by *A. actinomycetemcomitans* isolates to adhere and interact with epithelial cells may differ, possibly leading to differences in cell response, according to the main adhesion molecule enrolled in the interaction. Furthermore, the low expression of *omp100* and *omp29* by the serotype a strain indicates that recognition by the host, and its resultant inflammatory process, may be impaired when serotype a strains colonize, explaining association of this serotype with health.

The *A. actinomycetemcomitans* CagE is involved in host cell apoptosis (30). *cag*E transcripts were detected in JP2, independently on cell contact. This gene was not transcribed in the serotype a strain (ATCC 29523) in any of the studied conditions and was only detected in SUNY 465 after interaction with epithelial cells (Table 3).

Other genes such as *orf859*, associated with intracellular survival of *A. actinomycetemcomitans* (31) and *vapA*, similar with *Dichelobacter nodosus vap* (virulence-associated protein) (32), were also poorly transcribed by serotype a in most conditions when compared to the serotype b strains. However, transcription of both genes was strongly upregulated in ATCC 29523 after prolonged interaction with epithelial cells (Table 3).

Thus, *A. actinomycetemcomitans* presents mechanisms for recognition of environmental changes, which alters the transcription of genes needed for survival within the host. Some genes were upregulated in the presence of epithelial cells in all strains, such as *pga*, which encodes the synthesis of extracellular polysaccharide involved in biofilm formation (9). However, transcripts profiles largely differed among the strains, especially between serotype a (ATCC 29523) and serotype b (JP2 and SUNY 465). The transcription of genes encoding factors related to colonization such as *aae*, *flp*, *apaH*, and *pgA* and immune modulation such as *cdtB* was strongly upregulated after prolonged interaction with epithelial cells in serotype a. On the other hand, genes encoding immunogenic proteins associated with virulence such as *omp29* and *omp100*, host defense evasion (*ltxA*) and apoptosis (*cagE*) were poorly transcribed in serotype a when compared with both serotype b strains.

Strains belonging to the JP2 clone are genetically very homogeneous (33), although differences in their genomic content have been reported. On the other hand, JP2 and serotype b non-JP2 strains may present some differences in gene content (34). Furthermore, microarray data demonstrated that five operons were detected in *A. actinomycetemcomitans* with at least two genes with a two-fold difference or greater in expression levels between JP2 and non-JP2 genotypes in log grown cultures (34). However, the *ltx* operon was differentially expressed between JP2 and non-JP2 genotypes (34) as also shown here when strains JP2 and SUNY 465 were compared (Table 3). Furthermore, whilst *omp29* (*omp34*) was highly expressed by strain JP2 at the 2 hours control assay, this gene was also highly transcribed by one strain of the JP2 clone, but not by the others (34). On the other hand, genes differentially expressed between strain JP2 and non-JP2 serotype b strain (SUNY 465) as shown here, such as *omp100*, were not shown to be differentially expressed by Huang et al. (34). These differences should be attributed not only to different methods (microarray versus qPCR), but also to different studied conditions and tested strains.

Taken together, these data suggested that the regulation of virulence genes in *A. actinomycetemcomitans* can be considered strain-specific and contributes to the understanding of the large association of serotype b with aggressive periodontitis and serotype a with health.
